# Analysis of urban–rural differences in the relationship between grandparenting and the nutrition and health status of children aged 0–3 in China

**DOI:** 10.3389/fpubh.2024.1494222

**Published:** 2024-12-03

**Authors:** Lili Li, Zhenyu Zhang, Shili Tian, Xueting Shi

**Affiliations:** ^1^College of Humanities & Social Development, Northwest A&F University, Xianyang, China; ^2^Research and Evaluation Center of Rural Revitalization in Yellow River Basin, Northwest A&F University, Xianyang, China

**Keywords:** grandparenting, nutrition and health, urban, rural, China

## Abstract

**Background:**

The practice of grandparents taking on the role of primary caregivers for their grandchildren is widespread across both urban and rural regions in China. Yet, the existing body of research offered limited clarity on how grandparenting associated with nutrition as well as health of children aged 0–3 years, particularly in terms of potential differences between urban and rural areas in China. Therefore, this study aims to delve into the association between grandparenting and nutrition as well as health status of children aged 0–3 and its urban–rural differences in China.

**Methods:**

This study draws on data from 1,028 children aged 0 to 3 years and their families, collected through the China Family Panel Studies (CFPS) in 2020. Nutrition and health status of children aged 0–3 was assessed based on the indicators of malnutrition, medical consultations due to illness and developmental delay. Parental reports were used to determine the extent of grandparenting, which any caregiving arrangement involving grandparents, whether during daytime, nighttime, or both, were categorized as grandparenting. Multivariate regression analyses were conducted to explore the association between grandparenting and nutrition as well as health outcomes of children aged 0–3.

**Results:**

Regression analysis results indicated that grandparenting is positively associated with malnutrition (OR 1.323; 95% CI 1.008, 1.735), medical consultation for illness (OR 1.382; 95% CI 1.058, 1.804), and developmental delay (OR 1.502; 95% CI 1.508, 2.134) in children aged 0–3 years. The above association has been proven to be evident in rural settings, whereas it was not significant in urban environments.

**Conclusion:**

Grandparenting exhibits a negative association with nutrition and health status of children aged 0–3. It is imperative for parents to consider their children’s caregiving arrangements carefully, and foster nutrition and health status of children from birth to 3 years old actively.

## 1 Introduction

The foundational role of early nutrition and health in shaping an individual’s life trajectory cannot be overstated. Numerous studies have established a strong connection between childhood malnutrition, micronutrient deficiencies, developmental delay, and significant disease experiences with health outcomes in adulthood (1). These elements can profoundly influence brain structure and function, resulting in enduring adverse effects on cognitive and emotional well-being (2). In recent years, despite China’s rapid advancements in public health and medical technology, which have markedly enhanced the nutrition and health status of young children and significantly curtailed the prevalence of severe nutrition and health issues. For instance, the rate of developmental delay among children under the age of 5 was alarmingly high at 38% in 1992, yet it plummeted to just 1.1% by 2021 (3). Disparities still persist if benchmarking the developmental delay rates of Chinese children against those in developed nations (4). Consequently, the focus on the nutrition and health of young children remains a pressing concern.

Existing studies have elucidated that parenting patterns significantly influence nutrition and health status of young children. A survey in 2016 spanning parents of children aged 0 to 15 across over ten provinces in China revealed that 61.6% of families rely on grandparents for child-rearing support (5). Grandparenting seems to be a feature of Chinese parenting. As socio-economic development advances, the number of dual-income households has been on the rise (6). Given the inadequate availability of comprehensive childcare facilities in China (7), many families turn to the older generation to mitigate the caregiving burden faced by dual-income parents, hence the increasing prevalence of grand-parental care (8). The role of grandparents has gradually changed from auxiliary caregivers to primary caregivers (9). In China’s rural regions, the progression of urbanization compels young rural laborers, who are also parents, to migrate from the countryside to urban centers in pursuit of improved developmental opportunities. Consequently, their children are left to be raised by grandparents, giving rise to a phenomenon known as inter-generational caregiving in rural areas (10). Conversely, in urban locales, young parents encounter significant occupational stress and competition, with extended work hours and regular overtime becoming the standard practice (11). This situation necessitates familial reliance on internal support, particularly from grandparents, for child-rearing assistance.

As grandparenting gradually becomes a social norm, it has emerged as a significant topic of concern. However, the academic community has yet to reach a consensus on the definition of grandparenting. Sadruddin emphasized that the definition should encompass the frequency of interaction between grandparents and grandchildren, direct caregiving behaviors, as well as indirect forms of support (12). Some researchers also suggested that grandparenting essentially involves grandparents providing childcare for their grandchildren (13). However, it is important to note that while academic discussions have primarily focused on the content of grandparenting, the aspect of time spent on grandparenting has been largely overlooked. In reality, the care required by young children differs during the day and at night. Therefore, it is not sufficient to only consider the content of caregiving when discussing grandparenting; the allocation of parenting time must also be taken into account. With this in mind, this paper aims to further categorize grandparenting into daytime and nighttime patterns based on the time spent, and to explore the relationship between different allocations of grandparenting time and the nutrition and health of young children aged 0–3.

In the realm of research focusing on grandparenting, alongside the nutrition and health of young children, there is a predominant focus on the age groups of 0–6 and 6–12 years (5, 14), with a noticeable gap in studies concentrating on the crucial birth to 3-year-old. This early stage represents a pivotal period for optimal growth and development in young children, underscoring the importance of examining caregiving methods during these formative years. Moreover, when evaluating nutrition and health status of young children, prior researches has frequently employed WHO assessment standards, utilizing height and weight *Z*-scores to determine nutritional status, and relying on the frequency of illness or self-assessed health status as indicators of health (5, 15). While height and weight *Z*-scores did provide an accurate gauge of whether young children were on par with their peers’ growth trajectories, the concepts of malnutrition and developmental delay encompass a broader spectrum, touching upon the overall nutritional state of young children. In pursuit of a more objective and precise health measurement that truly reflects the actual health needs of young children, our study proposes the utilization of medical consultations due to illness as a more reliable health metric, as opposed to merely counting the frequency of illness or relying on self-assessed health statuses.

The body of research exploring “the relationship between grandparenting and the nutrition and health of young children” presented mixed findings. While some studies indicated a negative correlation, such as Jiang’s work, which suggested that grandparents, influenced by their own early experiences of poverty and limited health knowledge, may erroneously view obesity as sign of good health, thereby encouraging overeating with detrimental effects on the nutrition and health of young children (16). Conversely, other research posited that grandparenting can alleviate the home-work conflict for mothers, leading to increased family income and, consequently, greater investment in children’s health (13), which could positively impact young children’s nutrition and health. These varied conclusions, however, are shaped by the socio-economic backdrop of the families involved, with children from lower socio-economic backgrounds more prone to health challenges (17). Acknowledging the disparities in caregiving knowledge and socio-economic status, researchers have delved into the nuances of this relationship from an international perspective (12). This study aims to further dissect the relationship between grandparenting and the nutrition and health of children aged 0–3 through an urban–rural lens, considering the diversity of caregiving arrangements and enhancing the for assessing young children’s nutrition and health. This approach seeks to provide a more precise evaluation of how grandparenting influences the nutrition and health of young children.

Set against this context, the objective of this study is to delve into the dynamics between grandparenting and the nutrition as well as health status of 0–3 years old children, while also to shed light on the distinctions between urban and rural settings in this regard. The innovative contributions of this research are manifold: Firstly, it differentiates between daytime and nighttime care within the framework of grandparenting, aiming to elucidate the specific the association between these caregiving patterns and the nutrition and health of young children. Secondly, this study shifts the focus from the commonly researched age group of school-aged children (3–12 years old) to the less explored cohort of children aged 0–3 years, thereby venturing into relatively uncharted territory. Thirdly, this study adopts a broad spectrum of metrics to assess the nutrition and health outcomes of young children, including indicators of malnutrition, frequency of medical consultations due to illness, and instances of developmental delay. This multifaceted approach considerably bolsters the scientific integrity of the research and the trustworthiness of its findings. Lastly, acknowledging the distinctive urban–rural dichotomy in China, this paper undertakes an analysis of how the relationship between grandparenting and nutrition as well as health of young children varies across urban and rural landscapes. This investigation aims to provide a solid scientific foundation for the development of tailored intervention strategies.

## 2 Methods

### 2.1 Data source and study sample

The dataset employed in this study originates from the China Family Panel Studies (CFPS), a comprehensive and multidisciplinary social survey conducted in 2020 under the auspices of the Institute of Social Science Survey (ISSS) at Peking University. This ambitious project spanned across 25 provincial-level administrative regions, adopting a tripartite approach that encompasses individual, family, and community perspectives. Interviews were conducted with all members of the selected families, with parents providing responses for children under the age of 9.

**Figure 1:**
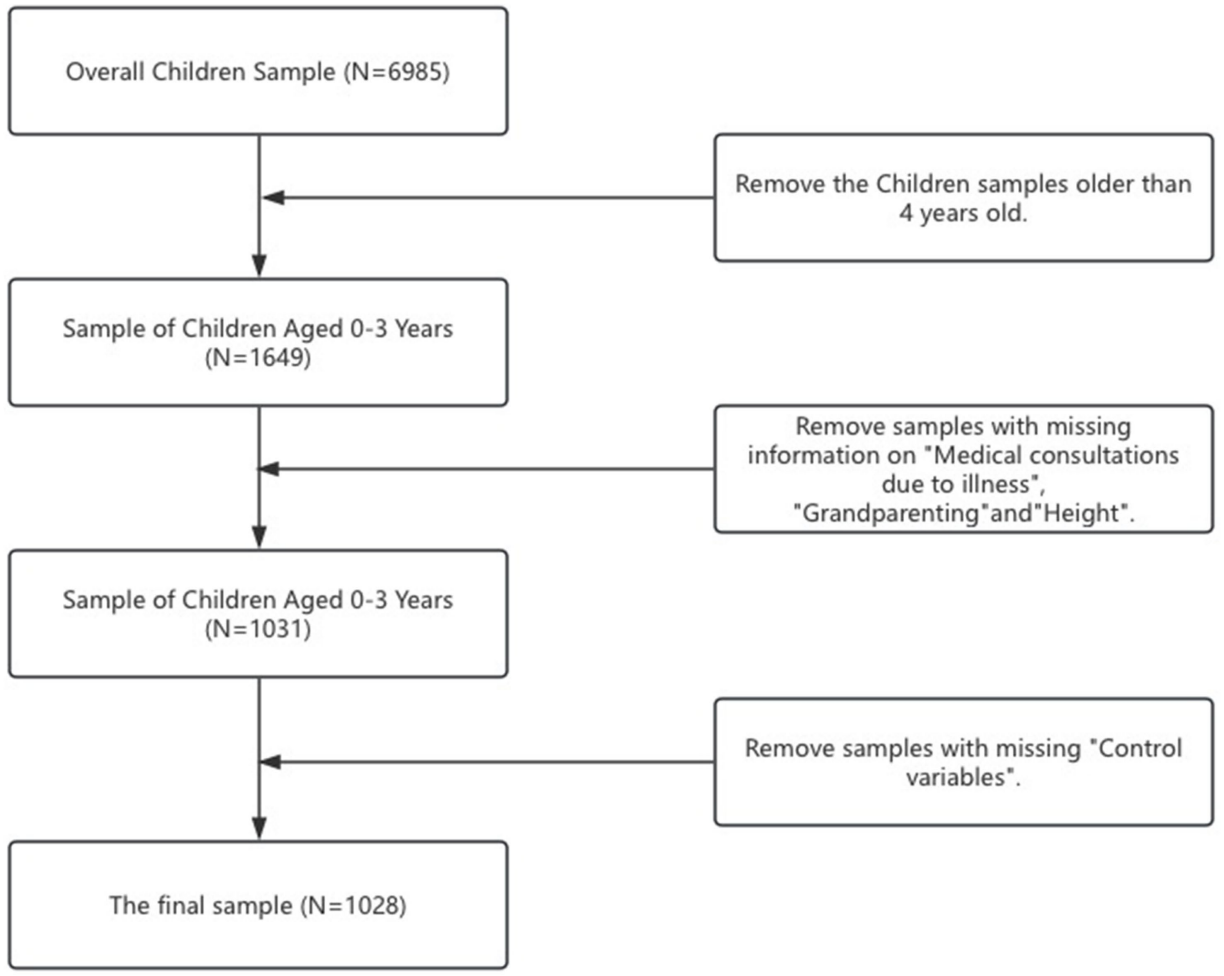
The Selection process of research sample.

For the purposes of this research, data were extracted from both the adult and young child questionnaires of the 2020 survey. The adult questionnaire gathered essential information about the family of the young child, whereas the young child questionnaire focused on aspects pertinent to the nutrition and health of young children. This paper processes the data as follows: For the purpose of analysis, sample children older than 4 years old were excluded from the total children sample (*n* = 6,958). Those who missed data of medical consultations due to illness (*n* = 342), those missing height data (*n* = 268), and those with grandparenting data missing (*n* = 8), as well as those missing control variables (*n* = 3), are excluded from the analysis. Consequently, the final sample included in the analyses comprised 1,028 children (Figure 1).^1^ Subsequently, the data pertaining to these young children were integrated with their corresponding family data, setting the stage for an in-depth analysis.

### 2.2 Variables

#### 2.2.1 Nutrition and health of young children

This study focuses on the nutrition and health status of children aged 0–3 years, employing three key indicators for measurement: malnutrition, medical consultations due to illness, and developmental delay status. The criteria for these measurements are as follows: Firstly, the malnutrition and developmental delay are calculated based on the World Health Organization’s (WHO) population height standards. Using the body height evaluation index of children aged 0–3 years to determine whether a child is experiencing malnutrition or developmental delay.^2^ Children identified as malnutrition or developmental delay are coded as 1, while those who are not are coded as 0. Secondly, medical consultations due to illness are derived from child questionnaire, where parents answered on behalf of their children aged 0–3 years: “Has the child visited a doctor due to illness in the past 12 months?” A visit to a doctor for illness is coded as 1; absence of such visits is coded as 0.

#### 2.2.2 Grandparenting

The core explanatory variable in this study is the grandparenting. Utilizing the China Family Panel Studies (CFPS) questionnaire designed for young children, the measurement indicators are as follows: “Who takes care of the child during the day?” and “Who takes care of the child at night?” Any caregiving arrangement involving grandparents, whether during daytime, nighttime, or both, is categorized as grandparenting. Conversely, all other caregiving setups are classified as non-grandparenting. For analytical purposes, grandparenting is coded as 1, whereas non-grandparenting receives a code of 0. Moreover, this study delved deeper by differentiating between daytime and nighttime grandparenting, based on responses from parents regarding whether grandparents are responsible for the child’s care during these periods.^3^

#### 2.2.3 Control variables

To delve into the familial contexts of the participants, this study meticulously selects control variables derived from the questionnaires focused on young children and their families. They include: The gender of the children within the age range of 0–3 years, is categorized as either female or male. The household registration status, distinguishing families based on urban or rural residency. The acquisition of commercial medical insurance. The educational attainment of the parents, segmented into two groups: those who have completed high school or above, and those who have not. The family’s economic situation, as subjectively evaluated by the parents. The timing of parenthood, specifically whether the parents had children later in their lives, which could influence parenting styles and resources available to the child.^4^ The frequency of spousal disagreements regarding inter-generational parenting practices, rated on a scale from never to very often (never/rarely/sometimes/often/very often).

### 2.3 Statistical analysis

Data cleaning and statistical analyses were performed using STATA 17.0 software (STATA Corp, College Station, Texas). The presentation of descriptive statistics includes the frequency (%) for categorical variables, along with the mean and standard deviation for continuous variables. To examine the disparities in grandparenting, nutrition and health, as well as personal and family characteristics of children aged 0–3 years between urban and rural settings, independent sample T-tests were employed. In investigating the relationship between grandparenting and the nutrition and health of children aged 0–3 years, the analysis controlled for crucial variables related to personal, parental, and family characteristics, while also incorporating regional fixed effects. This facilitated a multivariate logistic regression analysis aimed at elucidating the impact of grandparenting on the nutrition and health outcomes of young children. Additionally, the study employed multivariate logistic regression models to probe into the variations of these effects between urban and rural cohorts. The findings were primarily reported through odds ratios (OR), accompanied by the corresponding 95% confidence intervals (CI) and *p*-values. Statistical significance was determined at a threshold of *p* < 0.05, ensuring a rigorous assessment of the relationships under study.

## 3 Results

### 3.1 Sample children and family characteristics

The descriptive statistics of the relevant variables are presented in Table 1. The sample comprises 1,028 children aged 0–3 years, with the following characteristics observed: 42.02% were malnourished, 42.09% had medical consultations due to illness in the past year, and 18.29% were facing developmental delay. The proportion of grandparenting was 40.27% of the sample, of which 37.84% of children were grandparented at daytime, while 19.35% at nighttime. A majority of the children (64.78%) in rural areas, 54.86% children were male, and 22.86% children were covered by commercial medical insurance. Late parenthood was reported for 21.11% of fathers and 12.45% of mothers. 38.42% of fathers and 38.81% of mothers had attained high school or higher. The family’s economic condition average score was 2.409 (SD 1.222), with average scores for inter-generational and spousal parenting disagreements at 1.499 (SD 0.840) and 1.557 (SD 0.852), respectively.

**Table 1:** Descriptive statistical analysis based on urban–rural classification (*N* = 1,028).

Variables	All samples	U and R classification		*p*-value
		Urban (35.22%)	Rural (64.78%)	
Malnutrition				**0.003**
No	57.98%	64.09%	54.66%	
Yes	42.02%	35.91%	45.34%	
Medical consultation for illness				0.357
No	57.91%	58.84%	55.86%	
Yes	42.09%	41.16%	44.14%	
Developmental delay				0.224
No	81.71%	83.71%	80.64%	
Yes	18.29%	16.29%	19.36%	
Grandparenting				**0.000**
No	59.73%	50.56%	64.72%	
Yes	40.27%	49.44%	35.28%	
Grandparenting (day)				**0.000**
No	62.16%	52.77%	67.27%	
Yes	37.84%	47.23%	32.73%	
Grandparenting (night)				0.134
No	80.65%	83.15%	79.28%	
Yes	19.35%	16.85%	20.72%	
Child gender				0.549
Female	45.14%	46.41%	44.45%	
Male	54.86%	53.59%	55.55%	
Commercial medical insurance				**0.005**
No	77.14%	72.10%	79.88%	
Yes	22.86%	27.90%	20.12%	
Father’s late parenthood				**0.000**
No	78.99%	72.66%	82.29%	
Yes	21.11%	27.34%	17.71%	
Mother’s late parenthood				**0.049**
No	87.55%	84.81%	89.04%	
Yes	12.45%	15.19%	10.96%	
Father’s education level				**0.000**
High school and below high school	61.58%	38.40%	74.18%	
High school or above	38.42%	61.60%	25.82%	
Mother’s education level				**0.000**
High school and below high school	61.19%	37.30%	74.18%	
High school or above	38.81%	62.70%	25.82%	
Family economic status	2.409 ± 1.222	2.323 ± 1.189	2.456 ± 1.235	0.095
Frequency of inter-generational parenting disagreements (1 = never - 5 very often)	1.499 ± 0.840	1.433 ± 0.778	1.534 ± 0.870	0.058
Frequency of parenting disagreements between couples (1 = never - 5 very often)	1.557 ± 0.852	1.502 ± 0.781	1.587 ± 0.887	0.13

Data source: CFPS data (2020); *p*-value calculated by *t*-test. Bold font indicated that the results are significant.

When examining urban and rural differences, rural children had higher proportions of malnutrition, medical consultations due to illness, and developmental delay (45.34, 44.14, and 19.36%) compared to urban children (35.91, 41.16, and 16.29%). However, the proportion of grandparenting in urban settings (49.44%) was higher than that of rural areas (35.28%), and the daytime grandparenting for urban children (47.23%) also exceeded that for rural children (32.73%). Conversely, the proportion of nighttime grandparenting for rural children (20.72%) was higher than that for urban children (16.85%). The proportion of urban children with commercial health insurance (27.90%) was higher than that of rural children (20.12%). The proportion of late parenthood among urban parents (27.34% for fathers and 15.19% for mothers) was higher than that among rural parents (17.71% for fathers and 10.96% for mothers). 61.60% of urban fathers and 62.70% of urban mothers had a high school or above, compared to only 25.82% of rural parents; the average scores for family economic conditions were 2.456 (SD 1.235) for rural areas and 2.323 (SD 1.189) for urban areas; the average scores for inter-generational disagreement frequency and spousal disagreement frequency in urban areas were 1.433 (SD 0.778) and 1.502 (SD 0.781), while in rural areas, they were 1.534 (SD 0.870) and 1.587 (SD 0.887). There were significant differences between urban and rural areas in malnutrition (*t* = −2.936, *p* < 0.01), grandparenting (*t* = 4.460, *p* < 0.001), daytime grandparenting (*t* = 4.623, *p* < 0.001), commercial insurance (*t* = 2.846, *p* < 0.01), late parenthood (father: *t* = 3.633, *p* < 0.001; mother: *t* = 1.965, *p* < 0.05), parental education level (father: *t* = 12.019, *p* < 0.001; mother: *t* = 12.418, *p* < 0.001).

### 3.2 Association between grandparenting and child malnutrition

In the univariate regression analysis, Model 1 revealed a significant positive association between any form of grandparenting (OR 1.323; 95% CI 1.008, 1.735) and child malnutrition. Furthermore, several factors showed a significant negative association with malnutrition, these factors include the child’s gender (OR 0.756; 95% CI 0.585, 0.975), commercial health insurance (OR 0.722; 95% CI 0.528, 0.987), father’s late parenthood (OR 0.660; 95% CI 0.456, 0.954), and mother’s education level (OR 0.629; 95% CI 0.447, 0.885). Further analyses in Model 2 and Model 3 explored the association between different forms of grandparenting and child malnutrition. The findings highlighted that daytime grandparenting (OR 1.316; 95% CI 1.000, 1.730) was significantly positively association with child malnutrition. However, no significant association was found between nighttime grandparenting and child malnutrition, as detailed in Table 2.

**Table 2:** Logistic regression analysis of the association between grandparenting and malnutrition.

Dependent variable	Model 1		Model 2		Model 3	
	Malnutrition (All samples: 1028)		Malnutrition (All samples: 1028)		Malnutrition (All samples: 1028)	
	OR (95% CI)	*p*-value	OR (95% CI)	*p*-value	OR (95% CI)	*p*-value
Independent variable						
Grand-parenting	**1.323 (1.008, 1.735)**	**0.043**				
Grand-parenting (day)			**1.316 (1.000, 1.730)**	**0.049**		
Grand-parenting (night)					1.342 (0.972, 1.854)	0.074
Individual characteristics						
Genders	**0.756 (0.585, 0.975)**	**0.032**	**0.752 (0.582, 0.970)**	**0.029**	**0.759 (0.589, 0.980)**	**0.035**
Household registration	1.242 (0.924, 1.668)	0.150	1.242 (0.924, 1.669)	0.150	1.204 (0.896, 1.618)	0.216
Commercial medical insurance	**0.722 (0.528, 0.987)**	**0.042**	**0.723 (0.529, 0.989)**	**0.042**	**0.728 (0.532, 0.995)**	**0.047**
Parental characteristics						
Father’s late childbirth	**0.660 (0.456, 0.954)**	**0.027**	**0.661 (0.457, 0.957)**	**0.028**	0.663 (0.458 ，0.959)	0.029
Mother’s late childbirth	1.287 (0.822, 2.015)	0.268	1.288 (0.823, 2.016)	0.268	1.256 (0.803, 1.962)	0.317
Father’s education level	1.055 (0.752, 1.479)	0.755	1.056 (0.753, 1.480)	0.751	1.079 (0.770, 1.512)	0.656
Mother’s education level	**0.629 (0.447, 0.885)**	**0.008**	**0.631 (0.448, 0.887)**	**0.008**	**0.648 (0.463, 0.908)**	**0.012**
Family characteristics						
Family economic status	1.030 (0.928, 1.144)	0.569	1.032 (0.930, 1.146)	0.544	1.030 (0.928, 1.143)	0.570
Frequency of inter-generational parenting disagreements	0.996 (0.809, 1.227)	0.977	1.002 (0.814, 1.234)	0.980	1.005 (0.816, 1.237)	0.960
Frequency of parenting disagreements between couples	1.004 (0.818, 1.232)	0.969	1.000 (0.815, 1.227)	0.999	1.002 (0.816 ，1.229)	0.984

All regressions include area fixed effects; OR odds ratio, CI confidence interval. Bold font indicated that the results are significant.

### 3.3 Association between grandparenting and children’s medical consultations due to illness

In the analysis conducted within Model 4, the presence of any form of grandparenting (OR 1.382; 95% CI 1.058, 1.804) exhibited a significant positive association with children’s medical consultations due to illness over the past 12 months. Moreover, the possession of commercial medical insurance (OR 1.554; 95% CI 1.151, 2.098) and a higher frequency of parenting disagreements between couples (OR 1.302; 95% CI 1.063, 1.594) were also significantly associated with an increased number of medical consultations for children within the same time-frame. Further exploration in Model 5 and Model 6 investigates the association between different forms of grandparenting and children’s medical consultations. The findings indicated that daytime grandparenting (OR 1.399; 95% CI 1.069, 1.832) was significantly associated with an increased likelihood of medical consultations. However, nighttime grandparenting did not show a significant association with the frequency of children’s medical consultations, as detailed in Table 3.

**Table 3:** Logistic regression analysis of the association between grandparenting and medical consultation for illness.

Dependent variable	Mode 4		Model 5		Model 6	
	Medical consultation for illness (All samples: 1028)		Medical consultation for illness (All samples: 1028)		Medical consultation for illness (All samples: 1028)	
	OR (95% CI)	*p*-value	OR (95% CI)	*p*-value	OR (95% CI)	*p-*value
Independent variable						
Grand-parenting	**1.382 (1.058, 1.804)**	**0.018**				
Grand-parenting (day)			**1.399 (1.069, 1.832)**	**0.014**		
Grand-parenting (night)					1.038 (0.753, 1.432)	0.817
Individual characteristics						
Genders	1.087 (0.844, 1.399)	0.517	1.081 (0.839 ，1.392)	0.545	1.089 (0.846 ，1.401)	0.507
Household registration	1.172 (0.875, 1.569)	0.286	1.174 (0.877 ，1.573)	0.279	1.149 (0.859, 1.537)	0.349
Commercial medical insurance	**1.554 (1.151, 2.098)**	**0.004**	**1.555 (1.152, 2.100)**	**0.004**	**1.577 (1.169, 2.127)**	**0.003**
Parental characteristics						
Father’s late childbirth	1.024 (0.715, 1.466)	0.895	1.026 (0.717, 1.469)	0.885	1.043 (0.730, 1.492)	0.813
Mother’s late childbirth	0.935 (0.600, 1.456)	0.767	0.938 (0.602, 1.461)	0.778	0.890 (0.573, 1.383)	0.605
Father’s education level	0.801 (0.573, 1.119)	0.194	0.801 (0.573, 1.119)	0.194	0.816 (0.585, 1.139)	0.234
Mother’s education level	1.104 (0.790, 1.543)	0.560	1.102 (0.788, 1.541)	0.567	1.176 (0.844, 1.637)	0.337
Family characteristics						
Family economic status	0.966 (0.870, 1.072)	0.519	0.968 (0.872, 1.074)	0.542	0.971 (0.875, 1.077)	0.584
Frequency of inter-generational parenting disagreements	0.966 (0.786, 1.188)	0.750	0.972 (0.791, 1.195)	0.793	0.978 (0.796, 1.202)	0.838
Frequency of parenting disagreements between couples	**1.302 (1.063, 1.594)**	**0.011**	**1.297 (1.059, 1.588)**	**0.012**	**1.290 (1.054, 1.579)**	**0.013**

All regressions include area fixed effects; OR odds ratio, CI confidence interval. Bold font indicated that the results are significant.

### 3.4 Association between grandparenting and child developmental delay

Model 7 examines the association between any form of grandparenting and child developmental delay. The findings indicated a significant positive association between grandparenting (OR 1.502; 95% CI 1.058, 2.134) and the likelihood of child developmental delay. Additionally, the factors of mother’s late parenthood (OR 2.373; 95% CI 1.365, 4.127) and child gender (OR 1.436; 95% CI 1.025, 2.012) were significantly associated with an increased risk of child developmental delay. Conversely, commercial medical insurance (OR 0.538; 95% CI 0.339, 0.852), father’s late parenthood (OR 0.530; 95% CI 0.319, 0.881), and mother’s education level (OR 0.477; 95% CI 0.299, 0.760) showed a significant negative association with child developmental delay. Model 8 and 9 delved deeper into the distinctions between different forms of grandparenting associated with child developmental delay. The analyses revealed that both daytime (OR 1.473; 95% CI 1.034, 2.099) and nighttime (OR 2.023; 95% CI 1.369, 2.989) grandparenting were significantly and positively associated with child developmental delay, as detailed in Table 4.

**Table 4:** Logistic regression analysis of the association between grandparenting and developmental delay.

Dependent variable	Model 7		Model 8		Model 9	
	Developmental delay (All samples: 1028)		Developmental delay (All samples: 1028)		Developmental delay (All samples: 1028)	
	OR (95% CI)	*p*-value	OR (95% CI)	*p*-value	OR (95% CI)	*p*-value
Independent variable						
Grand-parenting	**1.502 (1.058, 2.134)**	**0.023**				
Grand-parenting (day)			**1.473 (1.034, 2.099)**	**0.032**		
Grand-parenting (night)					**2.023 (1.369, 2.989)**	**0.000**
Individual characteristics						
Genders	**1.436 (1.025, 2.012)**	**0.035**	1.420 (1.014, 1.989)	0.041	**1.465 (1.043, 2.056)**	**0.027**
Household registration	0.810 (0.548, 1.197)	0.291	0.806 (0.545, 1.192)	0.282	0.763 (0.515, 1.129)	0.177
Commercial medical insurance	**0.538 (0.339, 0.852)**	**0.008**	**0.539 (0.340, 0.854)**	**0.009**	**0.529 (0.333, 0.841)**	**0.007**
Parental characteristics						
Father’s late childbirth	**0.530 (0.319, 0.881)**	**0.014**	**0.529 (0.318, 0.880)**	**0.014**	**0.527 (0.318, 0.874)**	**0.013**
Mother’s late childbirth	**2.373 (1.365, 4.127)**	**0.002**	**2.377 (1.365, 4.139)**	**0.002**	**2.348 (1.355, 4.068)**	**0.002**
Father’s education level	0.777 (0.491, 1.231)	0.284	0.777 (0.490, 1.230)	0.282	0.815 (0.516, 1.288)	0.382
Mother’s education level	**0.477 (0.299, 0.760)**	**0.002**	**0.481 (0.302, 0.767)**	**0.002**	**0.472 (0.297, 0.750)**	**0.002**
Family characteristics						
Family economic status	1.058 (0.926, 1.210)	0.403	1.062 (0.929, 1.214)	0.376	1.054 (0.922, 1.205)	0.434
Frequency of inter-generational parenting disagreements	1.085 (0.834, 1.411)	0.543	1.094 (0.841, 1.424)	0.501	1.090 (0.837, 1.418)	0.520
Frequency of parenting disagreements between couples	1.024 (0.791, 1.325)	0.854	1.017 (0.786, 1.317)	0.894	1.028 (0.794, 1.332)	0.829

All regressions include area fixed effects; OR odds ratio, CI confidence interval. Bold font indicated that the results are significant.

### 3.5 The urban–rural differences in association between grandparenting and children’s nutrition and health

The regression outcomes of urban and rural sub-samples analysis were presented in Table 5. When focused on child malnutrition, the results reveal a significant positive association within the rural context for any form of grandparenting (OR 1.423; 95% CI 1.020, 1.984) and daytime grandparenting (OR 1.436; 95% CI 1.025, 2.011). However, these associations did not hold statistical significance within the urban sub-sample. Moreover, nighttime grandparenting did not show a significant association with child malnutrition in either urban or rural children. When examining medical consultations due to illness over the past 12 months, both any form of grandparenting (OR 1.478; 95% CI 1.060, 2.061) and daytime grandparenting (OR 1.479; 95% CI 1.056, 2.071) showed a significant positive association with such medical consultations among rural sub-sample. These associations, once again, were not observed to be significant within the urban cohort. Additionally, nighttime grandparenting did not exhibit a significant association with children’s medical consultations due to illness in either urban or rural settings. In the analysis centered on child developmental delay as the outcome variable, a significant positive association was identified in the rural sub-sample for any form of grandparenting (OR 1.547; 95% CI 1.012, 2.364), daytime grandparenting (OR 1.558; 95% CI 1.015, 2.390), and nighttime grandparenting (OR 2.101; 95% CI 1.315, 3.356). This pattern of association did not extend to the urban sub-sample.

**Table 5:** Logistic regression analysis of the association between grandparenting and the nutrition and health in rural and urban subsamples.

Dependent variable	Malnutrition											
	Rural		Urban		Rural		Urban		Rural		Urban	
	OR	*p*-value	OR	*p*-value	OR	*p*-value	OR	*p*-value	OR	*p*-value	OR	*p*-value
Grandparenting	**1.423**	**0.037**	1.239	0.386								
Grandparenting (day)					**1.436**	**0.035**	1.224	0.419				
Grandparenting (night)									1.451	0.059	1.205	0.551
Observations	666		362		666		362		666		362	

Dependent variable	Medical consultation for illness											
	Rural		Urban		Rural		Urban		Rural		Urban	
	OR	*p*-value	OR	*p*-value	OR	*p*-value	OR	*p*-value	OR	*p*-value	OR	*p*-value
Grandparenting	**1.478**	**0.021**	1.278	0.295								
Grandparenting (day)					**1.479**	**0.023**	1.345	0.210				
Grandparenting (night)									1.122	0.560	0.912	0.764
Observations	666		362		666		362		666		362	

Dependent variable	Developmental delay											
	Rural		Urban		Rural		Urban		Rural		Urban	
	OR	*p*-value	OR	*p*-value	OR	*p*-value	OR	*p*-value	OR	*p*-value	OR	*p*-value
Grandparenting	**1.547**	**0.044**	1.549	0.192								
Grandparenting (day)					**1.558**	**0.042**	1.430	0.292				
Grandparenting (night)									**2.101**	**0.002**	1.954	0.079
Observations	666		362		666		362		666		362	

All regressions include area fixed effects; OR odds ratio. Due to space limitations, CI was not presented, and all control variables used in regressions are the same as before. Bold font indicated that the results are significant.

## 4 Discussion

This study uses data from the China Family Panel Studies (CFPS) to explore the association between grandparenting and the nutrition and health outcomes of children aged 0–3 years. It uncovers that nationally, a substantial 40.27% of children within this age group are being parented by grandparents, with the rate climbing to 49.44% in urban settings and tapering to 35.28% in rural areas. In delving into the health and nutrition status of young children across three critical dimensions—malnutrition, medical consultations due to illness, and developmental delay—the analysis reveals a significant positive association between grandparenting and adverse outcomes in all three areas of children aged 0–3. Specifically, daytime grandparenting is linked to increased instances of malnutrition, more frequent medical consultations due to illness, and heightened risks of developmental delay. Similarly, nighttime grandparenting shows a positive association with child developmental delay. Moreover, when dissecting the data to compare urban versus rural disparities, it becomes evident that significant positive association between grandparenting and adverse outcomes of nutrition and health status of children aged 0–3 holds predominantly in rural families, spanning all three examined dimensions.

The findings align with a previous study by Yin Jun, utilizing CFPS data from 2018 (18). This consistency may stem from the fact that the parenting of children under the age of 3 still relies on families in China. Children typically begin kindergarten after turning 3 years old, highlighting the significant role of family caregiving practices during these early years. Research conducted by Bramlett has demonstrated that, even after adjusting for demographic factors, children raised by grandparents exhibit inferior physical and mental health outcomes compared to their counterparts raised directly by their parents (19). This discrepancy is attributed to the caregiving paradigms, capabilities, and approaches of grandparents. Evidence suggested that grandparents may not be well-versed in contemporary nutrition and health knowledge pertinent to child-rearing, with their outdated beliefs and methods detrimentally affecting the development and growth of the young (20, 21). Furthermore, grandparents are often found to be more lenient and potentially spoil the children, which can lead to adverse dietary and lifestyle habits (15, 22). Additionally, it is posited that the declining physical condition of aging grandparents could further limit their caregiving efficacy. These factors collectively underscore the complex dynamics at play within grandparenting arrangements and their implications for child well-being.

In this study, we define grandparenting as the involvement of grandparents in the caregiving of young children, with a distinction made between those providing primary care during daytime or nighttime hours. Our findings indicate that grandparents who solely provide care during the daytime are positively associated with increased instances of malnutrition, more frequent medical consultations due to illness, and developmental delay in children. Conversely, grandparents who only provide care at nighttime show a positive association with child developmental delay, while this association does not extend to medical consultations due to illness or malnutrition. The daytime represents a crucial period for children’s activities, exploration, and learning (23), necessitating greater supervision, interaction, and nutritional support. In contrast, nighttime primarily serves as a period for rest and sleep, with children typically retiring to bed earlier than adults, resulting in reduced activity levels and a lesser demand for caregiver engagement during these hours (24). Research shows that compared to parental care, children raised by grandparents are more susceptible to sleep disturbances and disorders at night, which can negatively impact their growth and health (25). This issue primarily arises because the “nighttime sleep guidance” process increases the caregiving pressure on grandparents (26). The physical challenges and emotional burdens can lead to negative emotions in grandparents, which are then transferred to the children during the “nighttime sleep guidance” process, adversely affecting their well-being. Consequently, the influence of grandparenting is more evident during daytime hours. Furthermore, within our sample, only 19.36% of grandparents were exclusive nighttime caregivers, a factor we attribute to the diminished impact of nighttime caregiving, possibly due to the limited sample size.

The association between grandparenting and nutrition as well as health outcomes of children aged 0–3 exhibits stark urban–rural disparities. This association is evident in rural settings, whereas it is not significant in urban environments. Research underscores the connection between an individual’s health and their living environment, highlighting the profound influence of China’s urban–rural divide on health outcomes (27). Several factors contribute to this disparity: Firstly, economic development in rural China is relatively behind, and infrastructure is less developed. In contrast, urban areas generally offer better living conditions and more comprehensive public health facilities (28), which are more conducive to children’s nutrition and health. Secondly, cultural differences between urban and rural areas significantly influence the parenting concept. Urban grandparents typically have higher expectations for childcare compared to their rural counterparts, as urban families have access to a wider range of social supports, such as professional parenting advice and parent–child activities (29). These urban parenting expectations extend beyond the basic needs of “ensuring adequate food and clothing” than their rural counterparts (21, 22). Such differences in parenting concepts can impact the quality of care and the nutrition and health levels of children.

Based on the main findings of this study, we propose the following two key recommendations: First, to ensure better care for young children, the government should actively establish accessible childcare centers and nurturing facilities, thereby reducing the reliance on grandparent caregiving. Furthermore, local health departments, community centers, or educational institutions can organize support groups focused on “grandparent caregiving” and provide free or low-cost parenting education courses to enhance grandparents’ childcare knowledge, indirectly improving children’s nutrition and health. Second, we recommend that the government, while advancing the national healthcare system, pay special attention to reducing the urban–rural disparity, particularly for low-income rural families who are not covered by medical insurance. For these groups, we suggest implementing mobile medical services to regularly visit rural communities, offering free child health check-ups, nutrition assessments, and consultations. For children facing nutritional and health challenges, the government should actively implement nutrition assistance programs to ensure they receive necessary nutritional support, thereby promoting their healthy growth.

Our study has some limitations: Firstly, the sample size is relatively small. Due to various reasons, there were some missing values in the original data. After excluding the missing values for key variables, the remaining sample size was limited. Secondly, there is a lack of long-term tracking. This study utilized the 2020 China Family Panel Studies, which conducts surveys biennially. However, this frequency is insufficient for supporting our long-term tracking research on the nutrition and health of children aged 0–3, making it difficult for us to accurately analyze the potential causal association between grandparenting and children’s long-term health status. If possible, in future research, we plan to conduct a long-term study on children aged 0–3 in China, and fully incorporate and consider different cultural and social background factors in the research process, to better assess causality in the association between grandparenting and child health outcomes. This approach will make the research on grandparenting more targeted, thereby enhancing the accuracy and applicability of the research findings.

## Data Availability

The original contributions presented in the study are included in the article/supplementary material, further inquiries can be directed to the corresponding author.

## Footnotes

^1^Referring to previous studies, in order to address the issue of missing samples, we regressed missing status on the core variables (grandparenting and household registration). The results show there is no significant association between grandparenting / household registration and missing status.

^2^To assess developmental delay and malnutrition, this study employs the World Health Organization (WHO) height standards. According to these standards, a child’s height that falls below the median of the age-specific height standard is classified as malnutrition. Furthermore, a height that is more than two standard deviations below the median of the age-appropriate height standard is indicative of developmental delay.

^3^For a detailed breakdown of the grandparenting sample, please refer to Table A1.

^4^In this context, the term “parents’ age at childbirth” denotes their age at the time of their infant’s birth. As per the globally accepted classification of childbearing age, conceiving children beyond the age of 35 is categorized as advanced maternal/paternal age.

## Appendix

**Table A1:** Distribution of the grandparenting sample.

	Grandparenting both day and night		Grandparenting during the day or night	
	Urban	Rural	Urban	Rural
Grandparenting (day)	–	–	118	97
Grandparenting (night)	–	–	8	17
Total Grandparenting	53	121	126	114

Data source: CFPS data (2020).
